# Fluoroscopic balloon dilation for early jejunojejunostomy obstruction after gastrectomy with roux-en-Y reconstruction: a case series of three patients

**DOI:** 10.1186/s40792-020-00871-4

**Published:** 2020-05-24

**Authors:** Teppei Kamada, Hironori Ohdaira, Sojun Hoshimoto, Satoshi Narihiro, Norihiko Suzuki, Rui Marukuchi, Hideyuki Takeuchi, Masashi Yoshida, Eigoro Yamanouchi, Yutaka Suzuki

**Affiliations:** 1 Department of Surgery, International University of Health and Welfare Hospital, 537-3, Iguchi, Nasushiobara, Tochigi, 329-2763 Japan; 2 Department of Radiology, International University of Health and Welfare Hospital, 537-3, Iguchi, Nasushiobara, Tochigi, 329-2763 Japan

**Keywords:** Gastrectomy, Early small bowel obstruction, Fluoroscopic balloon dilation, Barbed suture

## Abstract

**Background:**

Small bowel obstruction after gastrectomy with Roux-en-Y reconstruction (R-Y reconstruction) is not a rare complication. However, patients who need re-operation for this complication have a high rate of postoperative complications.

We report a case series of three patients who underwent fluoroscopic balloon dilation (FBD) for early jejunojejunostomy obstruction (JJO) after gastrectomy with Roux-en-Y reconstruction (R-Y reconstruction).

**Case presentation:**

Three patients were referred to our hospital for surgery for gastric cancer. Robot-assisted distal gastrectomy with D2 lymph node dissection and antecolic R-Y reconstruction were performed in two patients, and robot-assisted total gastrectomy with D1+ lymph node dissection and antecolic R-Y reconstruction was performed in one patient. The jejunojejunostomy was created as a side-to-side anastomosis using a linear 45-mm stapler. The entry hole was closed with a knotless barbed suture, and serosal-muscle layer suture reinforcement with an absorbable suture was performed at the jejunojejunostomy.

Subsequently, all the patients were diagnosed with JJO by computed tomography and upper gastrointestinal series. The average time to JJO from gastrectomy was 5 days (range 2–7); initial clinical symptoms were vomiting in all three cases, with simultaneous upper abdominal pain in one case. We successfully performed FBD in all three cases after unsuccessful conservative treatment using an ileus tube. The clinical symptoms improved soon after FBD, and all the patients were able to avoid re-operation. The average period to FBD from JJO was 10 days (range 4–14). The average procedure time was 46 min (range 29–68), and the average duration to oral intake from FBD was 4 days (range 2–5). The average duration of hospital stay after FBD was 12 days (range 9–15). There were no complications in any of the cases.

**Conclusion:**

FBD might be a feasible procedure to avoid surgery for early small bowel obstruction after gastrectomy with R-Y reconstruction.

## Introduction

Small bowel obstruction (SBO) after gastrectomy with Roux-en-Y reconstruction (R-Y reconstruction) is not a rare complication. Early small bowel obstruction (ESBO) within the first 30 postoperative days after the reconstructive surgery can be caused by technical problems, such as kinking, narrowing, or acute angulation of the anastomosis, while late SBO can occur secondary to internal hernia or intra-abdominal adhesions [1, 2].

Most patients diagnosed with ESBO after R-Y reconstruction are observed conservatively and do not require surgical intervention [1–4]. However, some patients need re-operation under general anesthesia for both diagnosis and treatment when their situation does not improve, and these cases usually have a high rate of postoperative complications [5, 6].

Recently, endoscopic or fluoroscopic balloon dilation has been performed to avoid re-operation for ESBO or Crohn’s disease, as it is minimally invasive and preserves intestinal length [7, 8].

We report three cases in which fluoroscopic balloon dilation (FBD) was successfully performed to avoid re-operation for early jejunojejunostomy obstruction (JJO) after gastrectomy with R-Y reconstruction.

## Case presentation

### Case 1

A 65-year-old man was referred to our hospital for surgery for advanced gastric cancer. Robot-assisted distal gastrectomy with D2 lymph node dissection and antecolic R-Y reconstruction was performed. The jejunojejunostomy was performed via minilaparotomy through an umbilical incision. The jejunojejunostomy was created 40 cm distal to the gastrojejunal anastomosis, as a side-to-side anastomosis using a linear 45-mm stapler (Autosuture, Covidien, Mansfield, MA, USA). The entry hole was closed with a knotless barbed suture (V-Loc^TM^ 180, Covidien, Mansfield, MA, USA). Serosal to muscle layer suture reinforcement was performed at the jejunojejunostomy using a 3-0 Vicryl suture (Ethicon, Somerville, NJ). Petersen’s defect and the jejunojejunal mesenteric defect were also closed with a 3-0 Vicryl suture during the surgical procedure. The pathological diagnosis was poorly differentiated adenocarcinoma, T4a, N3a, M0, stage IIIB.

The patient initially made good progress after the operation and started oral intake on postoperative day 1. However, on postoperative day 6, he complained of upper abdominal pain and vomiting. Computed tomography (CT) scans showed signs of jejunal dilation between the gastric remnant and jejunojejunostomy without strangulation. Insertion of an ileus tube for decompression and bowel rest was performed, but with no improvement. On postoperative day 10, FBD was performed. The procedure time was 68 min. On the day following FBD, his clinical symptoms improved. Oral intake was recommenced 4 days after FBD, and he was discharged 9 days after FBD.

He was followed up for 30 days and experienced no symptoms or other complications during this period.

### Case 2

A 75-year-old man was referred to our hospital for surgery for advanced gastric cancer. Robot-assisted distal gastrectomy with D2 lymph node dissection and antecolic R-Y reconstruction were performed. The jejunojejunostomy was created similarly as in case 1. The pathological diagnosis was moderately differentiated adenocarcinoma, T3, N1, M0, stage IIB.

On postoperative day 2, he complained of vomiting. CT scans showed signs of jejunal dilation between the gastric remnant and jejunojejunostomy without strangulation. Insertion of an ileus tube for decompression and bowel rest was performed, but with no improvement in his symptoms. Hence, FBD was performed on postoperative day 14. The procedure time was 29 min. His clinical symptoms improved on the day following FBD. Oral intake was commenced 5 days after FBD, and he was discharged 11 days after FBD.

He experienced no symptoms or other complications during the 60 days follow-up period.

### Case 3

A 79-year-old man was referred to our hospital for surgery for early gastric cancer. Robot-assisted total gastrectomy with D1+ lymph node dissection and antecolic R-Y reconstruction were performed. The jejunojejunostomy was created similarly as in case 1. The pathological diagnosis was moderately differentiated adenocarcinoma, T1b, N0, M0, stage IA.

**Fig. 1:**
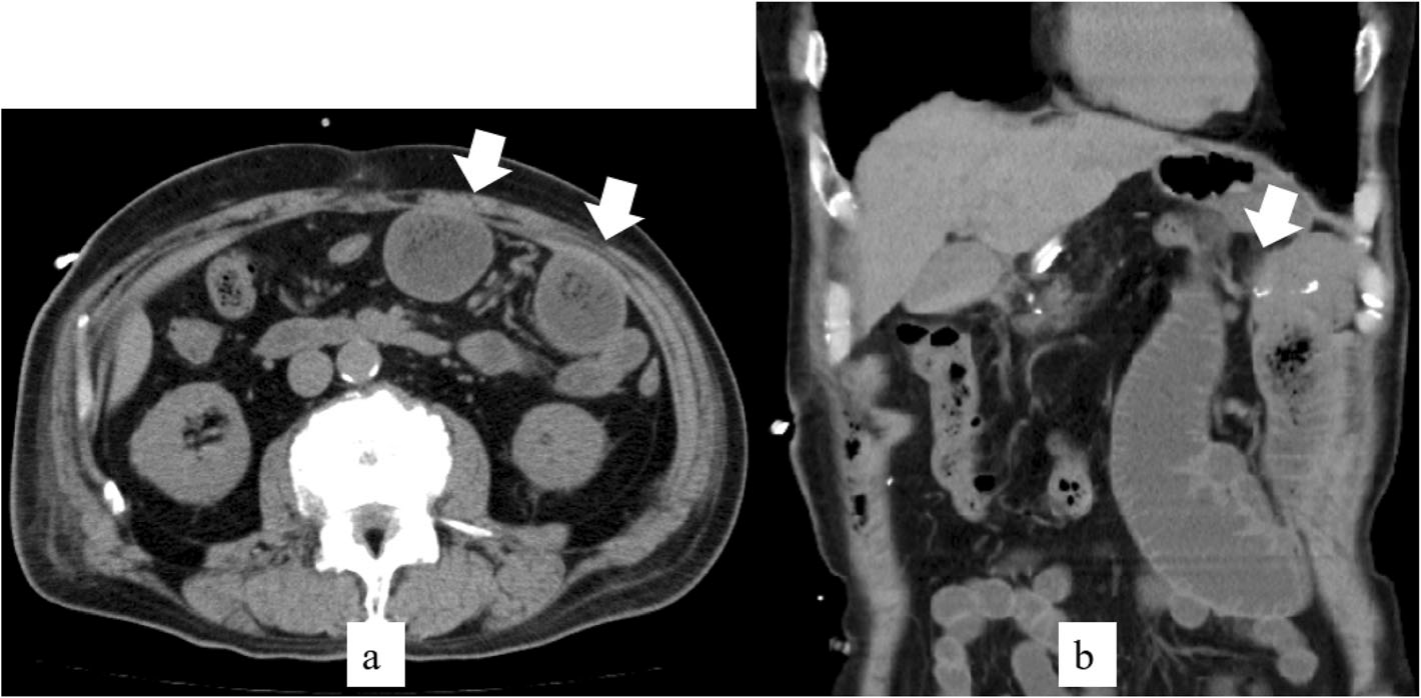
**a**, **b** CT scan imaging of jejunojejunostomy obstruction (imaging of case 3). **a** Dilation of the elevated jejunum (arrows). **b** Obstruction of the jejunojejunostomy (arrow)

On postoperative day 7, he complained of vomiting. CT scans showed signs of jejunal dilation between the esophagus and jejunojejunostomy without strangulation (Fig. 1a, b). Insertion of an ileus tube for decompression and bowel rest was performed, but without symptom improvement. Subsequently, FBD was performed on postoperative day 21. The procedure time was 42 min. His clinical symptoms improved on the day following FBD, and oral intake was commenced 2 days after FBD. He was discharged 15 days after FBD.

The patient experienced no symptoms or other complications during the follow-up period of 150 days after FBD.

The characteristics of the three patients with jejunojejunostomy obstruction are described in Table 1.

**Table 1:** Characteristics of the three patients with jejunojejunostomy obstruction after robot-assisted gastrectomy with Roux-en-Y reconstruction

Case	Age (years), sex	Gastrectomy Reconstruction	Pathological stage	Duration (days) (gastrectomy-JJO)	Symptoms	Duration (days) (JJO-FBD)	FBD time (minutes)	Duration (days) (FBD-oral intake)	Hospital stay (days) (FBD-discharge)	Complications
#1	65, M	RADG/D2 R-Y	IIIB (T4aN3aM0)	6	UAP, vomiting	4	68	4	9	None
#2	75, M	RADG/D2 R-Y	IIB (T3N1M0)	2	Vomiting	12	29	5	11	None
#3	79, M	RATG/D1+ R-Y	IA (T1bN0M0)	7	Vomiting	14	42	2	15	None

*RADG* robot-assisted distal gastrectomy, *RATG* robot-assisted total gastrectomy, *R-Y* Roux-en-Y reconstruction, *UAP* upper abdominal pain, *JJO* jejunojejunostomy obstruction, *FBD* fluoroscopic balloon dilation

### FBD procedure

**Fig. 2:**
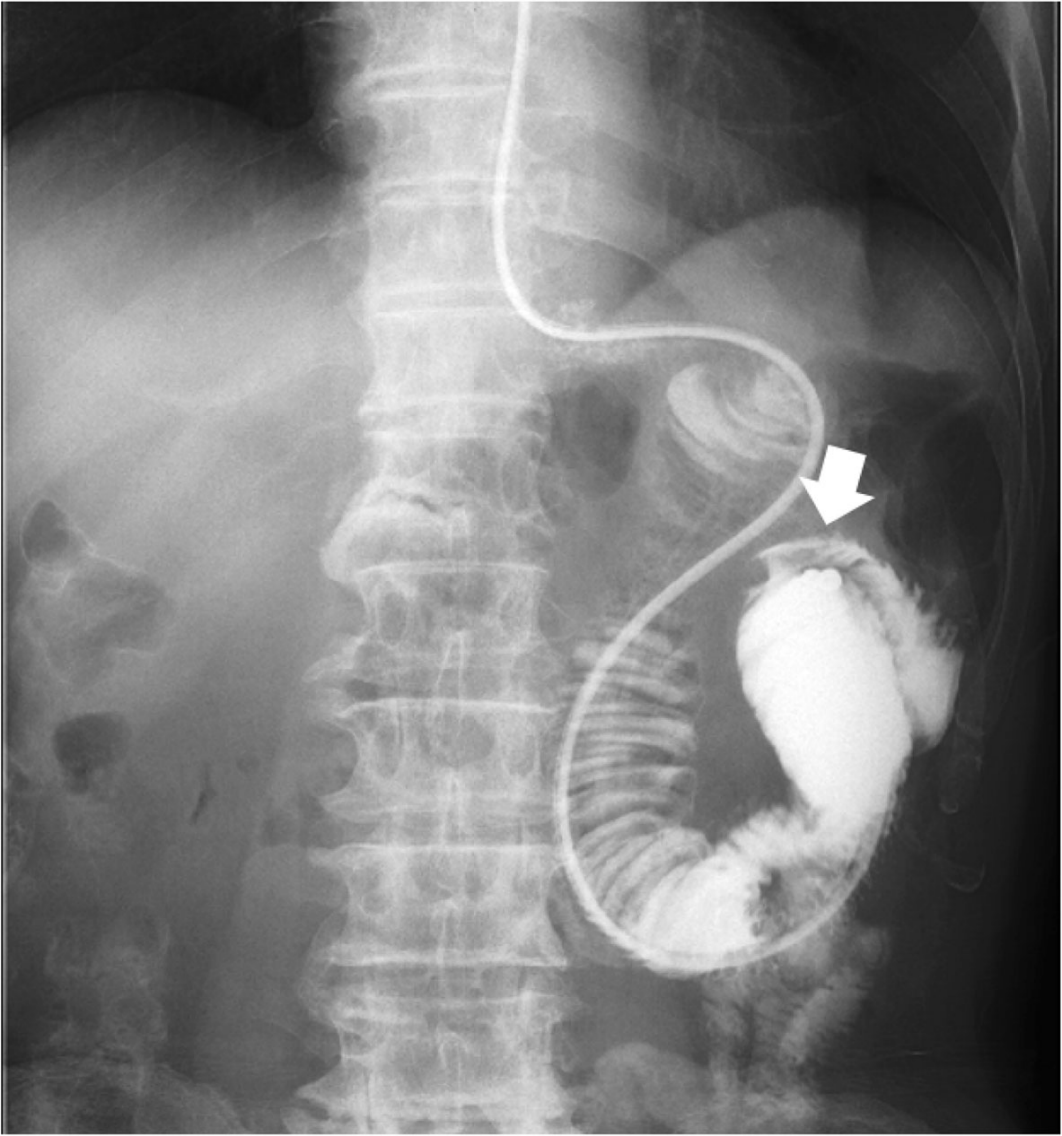
Upper gastrointestinal series through the ileus tube. Stenosis of the jejunojejunostomy (arrow)

Before FBD, the ileus tube was confirmed to be positioned just proximal to the stenosis. The location and length of the stenosis were identified by injection of contrast medium through the ileus tube (Fig. 2). The appropriate size of the balloon required was determined based on the severity of stenosis.

Flunitrazepam, 0.4 mg, was injected intravenously with the patient in the supine position.

**Fig. 3:**
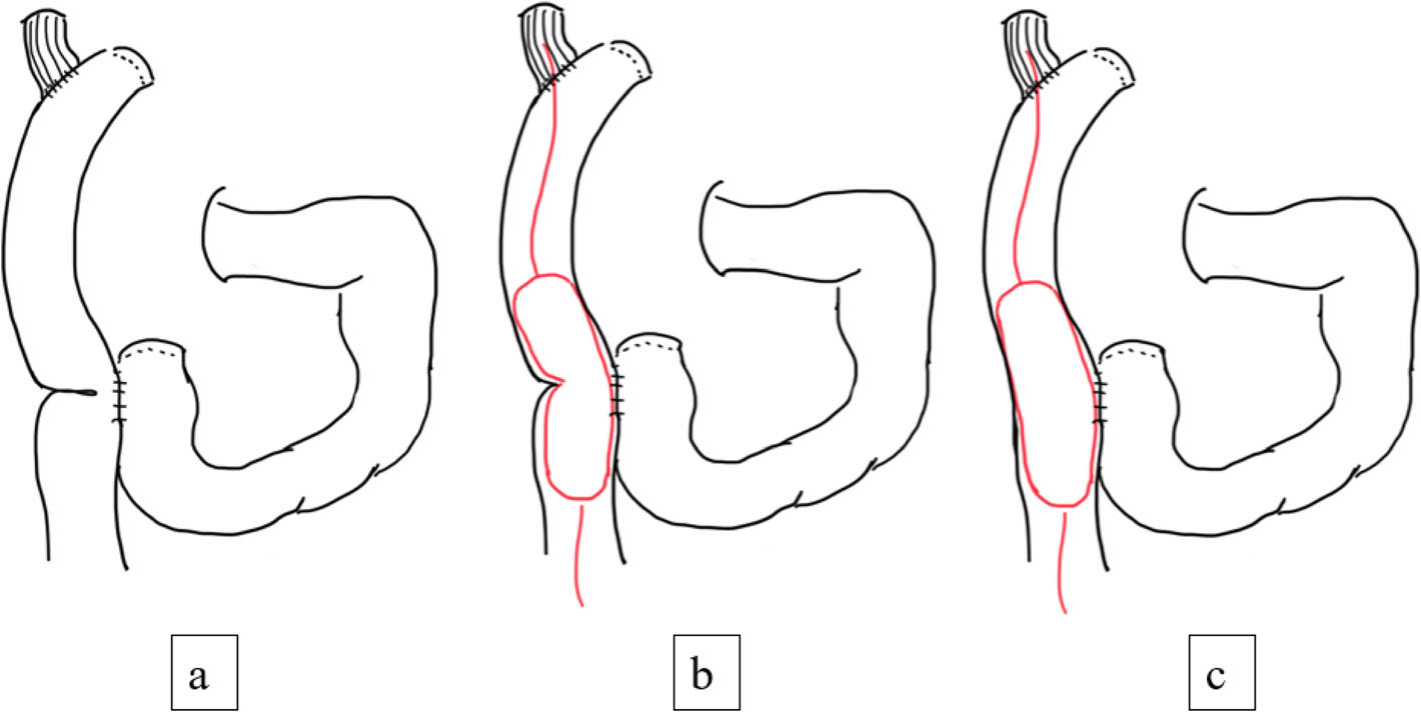
**a**, **b**, **c** Schema of fluoroscopic balloon dilation

The procedure was performed under constant fluoroscopic control (Fig. 3).

**Fig. 4:**
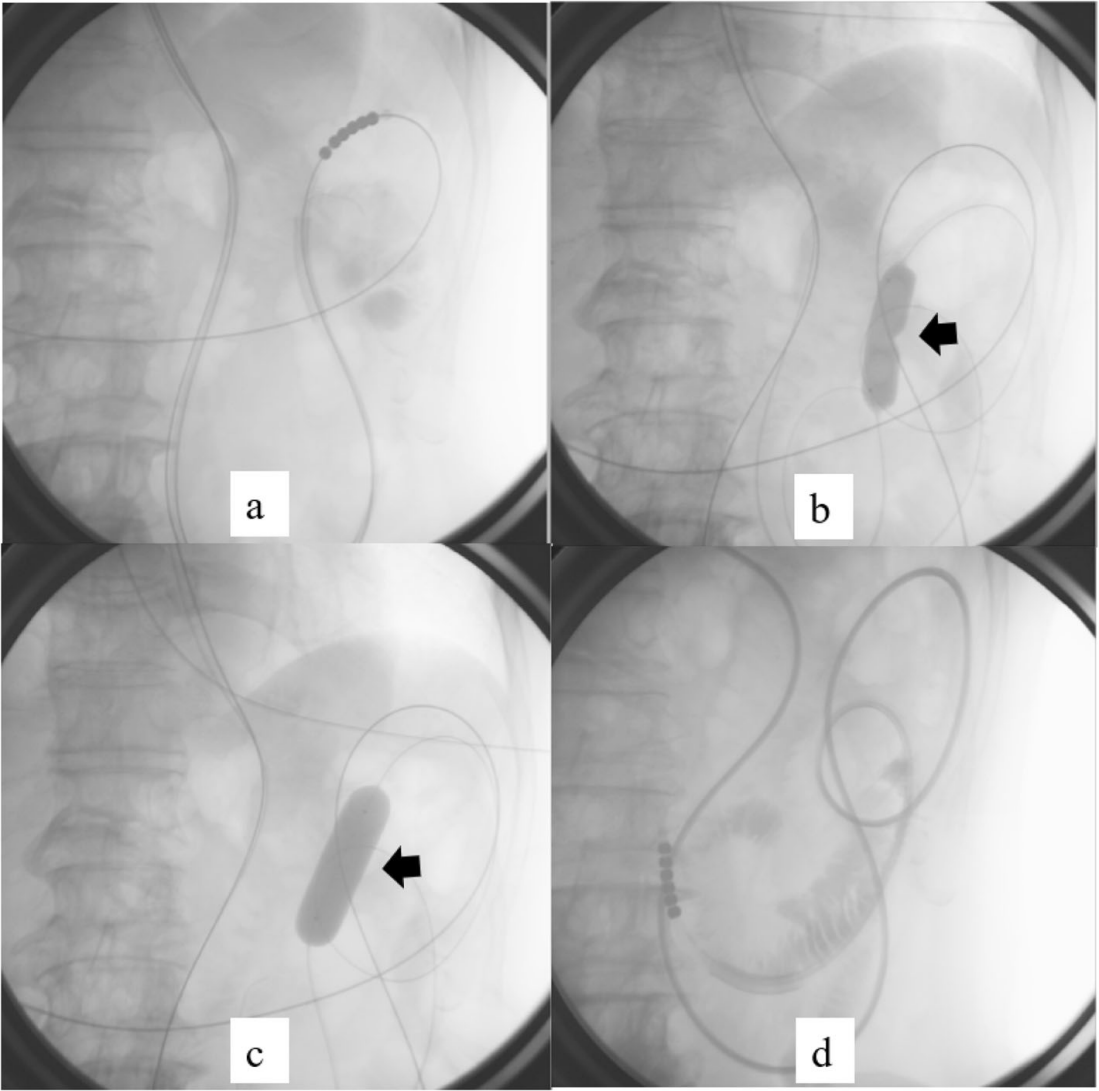
**a**, **b**, **c**, **d** Fluoroscopic balloon dilation procedure (imaging of case 3). **a** Radiograph showing insertion of the guidewire up to the site of stenosis. **b** Radiograph showing the “waist” caused by stenosis of the jejunojejunostomy (arrow). **c** Radiograph showing disappearance of the “waist” after balloon dilation (arrow). **d** Radiograph showing that the ileus tube could be passed across the site of stenosis

A 0.035-in, 260-cm-long stiff-angled hydrophilic guidewire (Radifocus, Terumo, Tokyo, Japan) was inserted through a nasal ileus tube and positioned over the location of the stenosis (Fig. 4a).

Next, a wire-guided balloon dilation catheter (CRE balloon catheter, Boston Scientific, Natick, MA, USA) was inserted up to the site of the stenosis. The balloon was gradually inflated with diluted water-soluble contrast medium under continuous fluoroscopic control until the stenosis completely disappeared, the inflation being maintained for 3 min thereafter (Fig. 4b, c). Then, diluted water-soluble contrast medium was injected through the catheter, and improvement of the stenosis was confirmed. Finally, the balloon catheter was removed, and an ileus tube was inserted through the guidewire to prevent re-stenosis after confirming that it could be passed through the site of stenosis (Fig. 4d). Postoperatively, the ileus tube was removed after confirming improvement of abdominal findings and bowel evacuation.

## Discussion

The overall reported incidence of SBO after laparoscopic gastrectomy with R-Y reconstruction is 2.6–3.6% [9, 10]. Half of the SBOs after R-Y reconstruction occur in the early postoperative period [2]. Although the definition of ESBO varied among investigators, it typically includes any clinically significant and radiologically proven partial or complete obstruction at the jejunojejunostomy site occurring within the first 30 postoperative days [3, 4].

ESBO is caused by several factors, such as kinking, narrowing, or acute angulation of the anastomosis, for which conservative treatment is inadequate and the patient needs re-operation in the form of either an open or laparoscopic procedure.

However, since early postoperative adhesions are often very dense and highly vascular, laparoscopic exploration often requires conversion to open laparotomy. Significant comorbidity caused by bowel resection or re-R-Y reconstruction also presented in at least half of the patients in previous studies [5, 6].

Recently, endoscopic or fluoroscopic balloon dilation has been shown to be a feasible procedure to avoid re-operation for ESBO or Crohn’s disease, as it is minimally invasive and preserves intestinal length [7, 8].

Additionally, it has the potential to delay the need for surgery and might be an alternative to surgery in patients who are not candidates or who wish to avoid or postpone surgery. Balloon dilation could also have many advantages over re-operation, including the absence of the need for general anesthesia, less pain, faster return of bowel function, shorter hospital stay, fewer complications, lower cost, and no risk of further adhesion formation [7, 8].

Additionally, FBD has several advantages over endoscopic balloon dilation. Although endoscopic balloon dilation is very useful for the treatment of proximal SBO, double-balloon enteroscopy is necessary to reach the stenosis in cases of distal SBO, which is difficult to insert. Further, not all institutions have double-balloon enteroscopy systems. FBD, on the other hand, can be easily performed for distal SBO. Another advantage is that FBD is more comfortable and less invasive for patients than endoscopic balloon dilation because of the size of the catheter device.

Tsuao et al. suggested that FBD might be safe and effective for treating postoperative non-anastomotic strictures of the proximal small bowel. In their study, of 44 patients, technical success with FBD was achieved in 39 (88.6%) patients, and the clinical success rate was 87.2%. There were no major complications directly related to FBD [7].

Baars et al. reported a systematic review of 13 studies, including a total of 310 patients, in whom double-balloon enteroscopy for small bowel strictures was performed. As a result, surgery was avoided in 80% of the patients. After the first dilatation, 46% were treated with re-dilatation and only 17% required surgery. The complication rate was 4.8% per patient and 2.6% per dilatation. This included five patients with perforations, three patients with acute pancreatitis, one patient who suffered from hemorrhage that required blood transfusions, three patients with hyperamylasemia, and three patients in whom the complications were unknown [8].

**Fig. 5:**
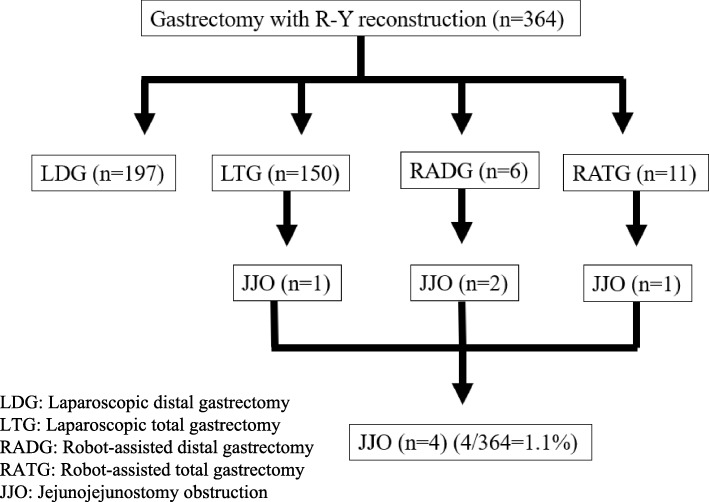
Incidence of jejunojejunostomy after gastrectomy with Roux-en-Y reconstruction at our hospital

Evaluation of the surgeries performed at our hospital showed that JJOs occurred in 1.1% (4/364) patients after gastrectomy with R-Y reconstruction (including LDG, LTG, RADG, RATG) (Fig. 5).

During re-operation after LTG, the cause of JJO after LTG was found to be adhesions between the jejunojejunostomy staple line and abdominal tissue. We consider that the cause of JJOs in our three cases were closure of the entry hole of the jejunojejunostomy with a knotless barbed suture and serosal to muscle layer suture reinforcement of the jejunojejunostomy. This type of closure probably resulted in excessive kinking of the jejunojejunostomy. We recently commenced using barbed sutures in order to reduce surgical duration. Barbed sutures offer a fast, secure, and effective alternative to conventional suture repair during laparoscopic surgery. However, cases of surgical complications, such as SBO, associated with the use of barbed suture devices have been reported [11, 12]. Subsequently, since we encountered these three cases of JJOs over a short period of time, we reinstituted the traditional closure method of the entry hole using only continuous 3-0 Vicryl sutures. We have not encountered JJOs since then.

In a recent study of 1097 patients who underwent laparoscopic R-Y gastric bypass [3], early JJO occurred in 13 patients (1.2%). In that report, the average time to presentation was 15 days (range 5–27). Patients presented with a combination of nausea, vomiting, and abdominal pain; all underwent CT to confirm the diagnosis. Ten patients (77%) were treated conservatively and three (23%) required operation with the creation of a new jejunojejunostomy between the distal Roux limb and proximal common channel to bypass the narrowed jejunojejunostomy.

In our three cases, all patients could be diagnosed by CT and upper gastrointestinal series. The average interval from gastrectomy to JJO was 5 days (range 2–7), initial clinical symptoms were vomiting in all three patients, and simultaneous upper abdominal pain in one patient. These results were similar to recent reports.

We conducted FBD in all three cases after unsuccessful conservative treatment using an ileus tube.

FBD resulted in rapid improvement in clinical symptoms, and all patients were able to avoid re-operation. The average period to FBD from JJO was 10 days (range 4–14). The average procedure time was 46 min (range 29–68), and average duration to oral intake was 4 days (range 2–5), The average hospital stay following FBD was 12 days (range 9–15). There were no complications. The longer procedure time (68 min) in case 1 was due to a longer time to guidewire passage.

Existing guidelines recommend that trials of non-operative management should not exceed 3–5 days because the likelihood of spontaneous resolution decreases substantially after this period [13, 14]. However, in our experience, FBD was useful after conservative treatment for 14 days, suggesting that there might be no restriction on the timing for FBD for SBO in the absence of findings suggestive of complete obstruction, strangulation, or necrosis.

The limitations of our research are that it included only few cases and only short-term follow-up was performed.

However, our experience suggests that FBD could be an alternative procedure to avoid surgery for ESBO after gastrectomy with R-Y reconstruction.

## Conclusion

FBD could be a feasible procedure to avoid surgery for ESBO after gastrectomy with R-Y reconstruction.

## Data Availability

Data sharing is not applicable to this article as no datasets were generated or analyzed during the current study.

## Abbreviations

SBO: Small bowel obstruction
R-Y reconstruction: Roux-en-Y reconstruction
ESBO: Early small bowel obstruction
FBD: Fluoroscopic balloon dilation
JJO: Jejunojejunostomy obstruction
LDG: Laparoscopic distal gastrectomy
LTG: Laparoscopic total gastrectomy
RADG: Robot-assisted distal gastrectomy
RATG: Robot-assisted total gastrectomy
